# Toward Image–Magnetic Flux Leakage Dual-Modality Inspection of Steel Pipes and Curved Steel Surfaces: Evidence, Fusion Strategies, and Robotic Acquisition

**DOI:** 10.3390/s26216850

**Published:** 2026-10-29

**Authors:** Bin Huang, Guofeng Du, Hongqiang Yuan

**Affiliations:** 1 School of Electronics and Information, Yangtze University, Jingzhou 434023, China; 2023730072@yangtzeu.edu.cn; 2 School of Urban Construction, Yangtze University, Jingzhou 434023, China; gfdu@yangtzeu.edu.cn

**Keywords:** steel pipe surface defect, magnetic flux leakage, machine vision, multimodal fusion, deep learning, transformer, wall-climbing robot, nondestructive testing, defect quantification

## Abstract

**Highlights:**

**What are the main findings?**

- The review organizes 126 works (2016–2026) into four themes (vision, MFL, fusion, and robotic platforms) and compares fusion strategies at the data, feature, and decision levels.
- When results are synthesized by evaluation protocol, vision detectors reach 82–83% mAP@0.5 on public benchmarks and up to 97.9% on self-built datasets, while strict-IoU scores fall to ≈47% within a single study and MFL baselines on the PipeMFL-240K benchmark reach mAP@0.5 =0.498.

**What are the implication of the main finding?**

- Within the search scope, no study has shown that spatially registered image–MFL fusion outperforms matched single-modality baselines.
- Cross-device registration, paired-data scarcity, and quantification accuracy are the main obstacles; physics-informed Transformer fusion and the joint design of sensor placement and robot motion are identified as priority directions.

**Abstract:**

Steel pipes and curved steel plates in the petroleum, chemical, and energy industries are prone to surface and weld defects that threaten structural integrity. Single-modality inspection by machine vision or magnetic flux leakage (MFL) struggles under real production conditions, including uneven illumination, occlusion, and electromagnetic noise. This review examines how far published evidence (2016–2026) supports combined image–MFL inspection, organizing 126 works into four themes (vision, MFL, fusion, and robotic platforms) and positioning them against fifteen prior surveys. Reported results, synthesized by dataset and evaluation protocol, show that vision detectors reach 82–83% mAP@0.5 on public benchmarks and up to 97.9% on self-built datasets, strict-IoU scores fall to ≈47% within a single study, and MFL baselines on the large-scale public PipeMFL-240K benchmark reach mAP@0.5 =0.498. Fusion evidence is graded by comparison design. Data-, feature-, and decision-level fusion schemes are compared; bidirectional cross-modal guidance is proposed for future testing. Cross-device registration, paired-data scarcity, quantification accuracy, and inconsistent evaluation are the main obstacles; physics-informed Transformer fusion and joint sensor–robot design are promising directions.

## 1. Introduction

Steel pipes and curved steel plate structures are fundamental components in petroleum and natural gas transportation, chemical processing, power generation, and offshore engineering. During manufacturing, forming, welding, transportation, and service, various surface and near-surface defects inevitably appear, including cracks, scratches, pits, inclusions, laps, weld undercuts, and corrosion [1,2,3]. If such defects are not detected before anti-corrosion coating or commissioning, they may evolve into leakage or rupture accidents, causing severe economic losses and safety hazards. Consequently, surface defect inspection has become a critical step in quality control in the steel industry, and the demand for automated, objective, and high-precision inspection continues to grow [4,5,6].

**Figure 1:**
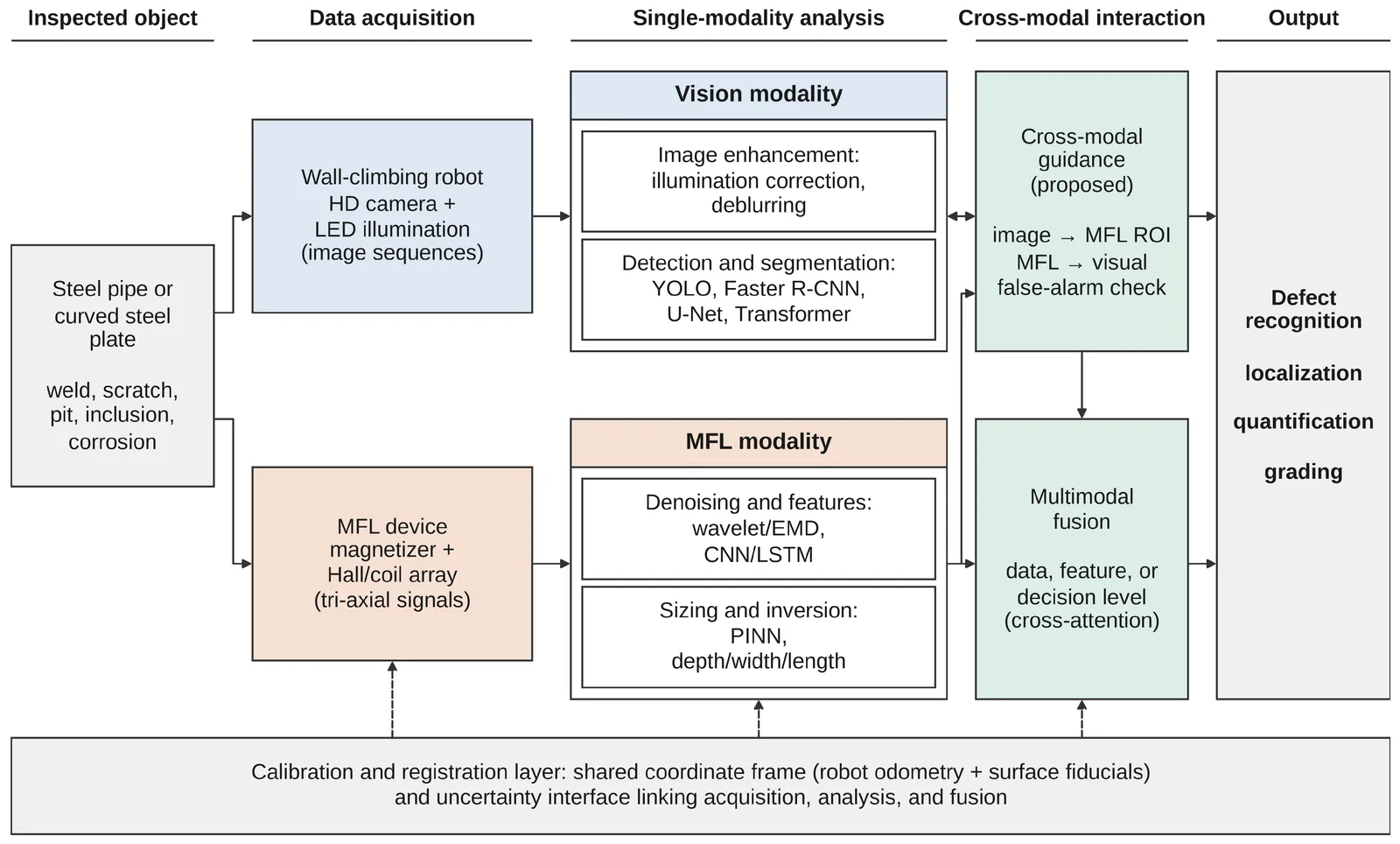
Conceptual framework for image–MFL dual-modality inspection of steel pipes and curved steel structures. Robot-borne visual and MFL acquisition feeds single-modality analysis, bidirectional cross-modal guidance (proposed), and data-, feature-, or decision-level fusion, all resting on a shared calibration and registration layer.

Traditional inspection relies on manual visual examination and conventional nondestructive testing (NDT) techniques such as magnetic particle testing, penetrant testing, ultrasonic testing (UT), eddy current testing (ECT), and magnetic flux leakage (MFL) testing [7,8,9]. Every single technique, however, has intrinsic limitations. Machine vision provides intuitive morphology, rich texture, and precise localization, yet it is easily disturbed by uneven illumination, rust, water stains, welding spatter, and foreign-object occlusion, and it is blind to subsurface defects [10,11]. MFL testing, in contrast, is highly sensitive to surface and near-surface discontinuities in ferromagnetic materials, requires no coupling agent, and tolerates harsh environments; however, MFL signals do not directly depict defect morphology; localization accuracy is limited, and the discrimination of compound defects remains difficult [9,12,13]. These complementary characteristics motivate a dual-modality approach in which image and MFL information are jointly acquired, mutually guided, and deeply fused [14,15,16]. Figure 1 outlines this approach: a wall-climbing robot acquires visual images while a magnetizer with a sensor array records MFL signals; after single-modality analysis, the two modalities interact through cross-modal guidance and are fused at the data, feature, or decision level to support defect recognition, localization, quantification, and grading. A calibration and registration layer, providing a shared coordinate frame and an uncertainty interface, underpins all three stages.

Meanwhile, the hardware carriers of inspection sensors have evolved rapidly. Magnetic-adhesion wall-climbing robots and pipeline in-line inspection robots can now carry high-definition cameras, illumination systems, and MFL probes onto vertical walls, curved surfaces, and pipe interiors, enabling full-coverage automatic scanning of large steel structures such as storage tanks, pressure vessels, hydraulic gates, ship hulls, and long-distance pipelines [17,18,19,20]. The convergence of robotic acquisition, deep learning, and multimodal fusion is reshaping the field from offline, laboratory-scale analysis toward online, field-deployable intelligent inspection systems [21,22,23].

Existing surveys cover visual inspection [1,2,3,4,5,6,10,11], MFL interpretation [9,13,24], pipeline inspection technologies and robots [25,26,27], and broad NDT fusion [15]. This review connects these areas through study-level evidence on camera–MFL pairing, matched-baseline comparisons, spatial registration, and acquisition constraints; Table 1 positions it against fifteen prior surveys, including four published in 2025–2026. The contributions of this review are fourfold:

(1) A four-theme taxonomy of 126 representative works (2016–2026), identified through a database search with PRISMA-style accounting and a targeted query extension (Section 2) and positioned against fifteen prior surveys, including four from 2025 to 2026.
(2) A comparison of reported detection and sizing results by dataset, evaluation metric, split, and inspection setting.
(3) Fusion evidence is graded into controlled comparisons, system-level changes, capability demonstrations, and fusion failures, and the requirements for testing registered image–MFL fusion are stated.
(4) The analysis shows how robotic acquisition constraints (the registration error budget, magnetizer–payload interference, and crawler speed) limit the fusion level achievable in the field.

The remainder of this review is organized as follows. Section 2 describes the review methodology and corpus statistics. Section 3 and Section 4 review vision-based and MFL-based detection, respectively. Section 5 analyzes fusion strategies and grades the fusion evidence. Section 6 covers robotic acquisition, open challenges, and future directions, and Section 7 concludes the review.

## 2. Review Methodology and Statistical Landscape

**Figure 2:**
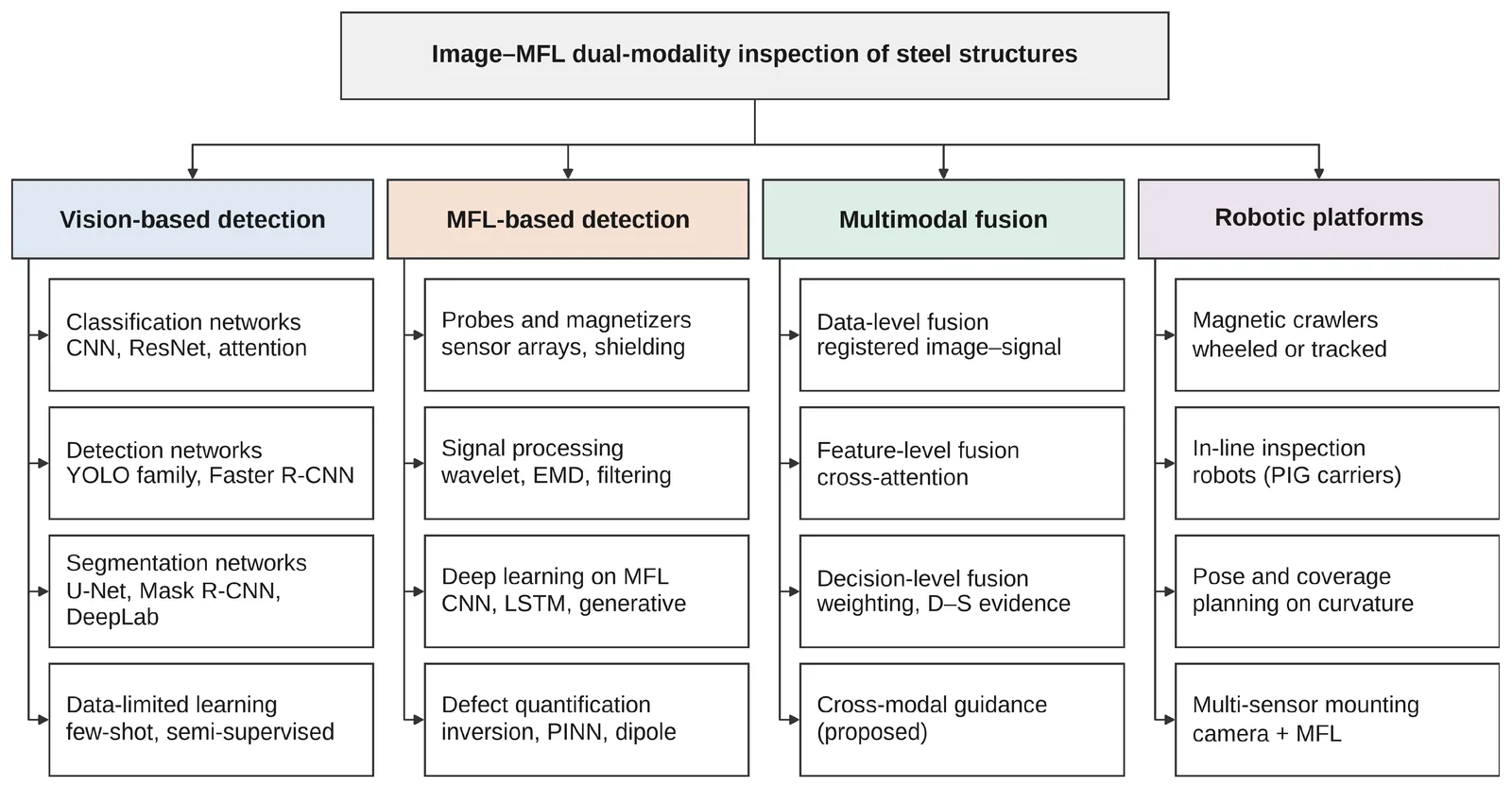
Taxonomy of the methods reviewed in this paper, organized into four themes: vision-based detection, MFL-based detection, multimodal fusion, and robotic acquisition platforms.

This section describes how the corpus was assembled and characterized. Records from two bibliographic databases and a supplementary Google Scholar search were deduplicated and screened in stages (Section 2.1), and the retained works were grouped into four themes (vision-based detection, MFL-based detection, multimodal fusion, and robotic acquisition platforms), as shown in Figure 2. Section 2.2 then profiles the corpus by year and theme.

### 2.1. Search Strategy and Screening Procedure

**Figure 3:**
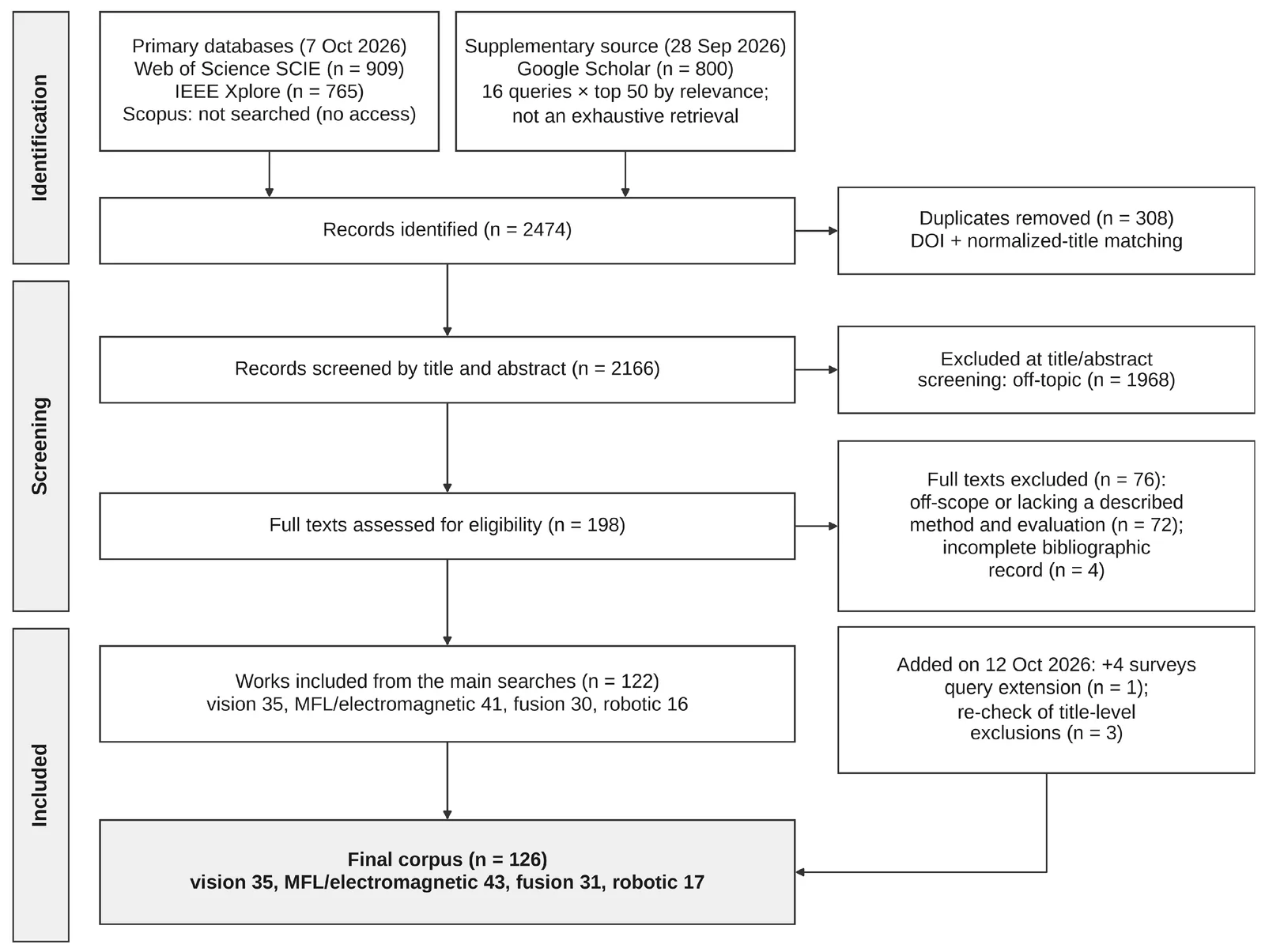
PRISMA-style flow of the literature identification, screening, and inclusion procedure. Database searches were executed on 7 October 2026 (Web of Science Core Collection, IEEE Xplore) and 28 September 2026 (Google Scholar, supplementary: 16 queries, top 50 records each by relevance); on 12 October 2026, a targeted query extension (one survey) and a re-check of title-level exclusions (three surveys) added four surveys.

The literature search followed a PRISMA-style procedure (Figure 3). The Web of Science Core Collection (SCIE) and IEEE Xplore served as the primary databases and were searched on 7 October 2026 over the publication window January 2016 to October 2026; Google Scholar, which also indexes Chinese-language publications, was searched on 28 September 2026 as a supplementary source. Scopus was not searched because institutional access was unavailable. English queries combined object terms (“steel pipe”, “steel surface”, “steel plate”, “pipeline”, “weld”) with method terms (“defect detection”, “defect recognition”, “magnetic flux leakage”, “machine vision”, “multimodal”, “sensor fusion”, “wall-climbing robot”, “in-line inspection”, “deep learning”). The full Web of Science topic query was TS = ((“steel pipe” OR “steel surface” OR “steel plate” OR “steel strip” OR pipeline* OR weld* OR “curved steel”) AND (“defect detection” OR “defect recognition” OR “flaw detection” OR “surface defect*”) AND (“machine vision” OR “deep learning” OR “magnetic flux leakage” OR “MFL” OR multimodal* OR “sensor fusion” OR “data fusion” OR “wall-climbing robot” OR “climbing robot” OR “in-line inspection”)) AND PY = (2016–2026) AND LA = (English), and an equivalent all-metadata query was used on IEEE Xplore. Because Google Scholar does not support long nested Boolean strings, it was queried with 16 short keyword strings (12 English and 4 Chinese, covering vision, MFL and eddy-current methods, multimodal fusion, and climbing robots), with publication years restricted to 2016–2026, and the top 50 ranked records for each query were exported. Its approximate hit counts were not recorded, so this component is a relevance-ranked sample; reproducibility rests on the Web of Science and IEEE Xplore searches. On 12 October 2026, a targeted Web of Science query that added “inspection” and “sizing” to the defect block identified one survey missed by the initial strings [24], and a re-check of title-level exclusions recovered three surveys that had been screened out because their titles contain no defect term [25,26,27]; the record count of the extension query was not recorded. All four surveys are included in the corpus and in Table 1. The full database-specific strings and all 16 Google Scholar queries are listed in Supplementary Note S1.

The searches yielded 909 records from Web of Science and 765 from IEEE Xplore, together with 800 Google Scholar records (16 queries × 50), for 2474 in total. Records were included if they (i) address steel pipes, plates, welds, strips, or comparable curved ferromagnetic components; (ii) propose or evaluate a vision-based, electromagnetic, or fused sensing and interpretation method, or a robotic acquisition platform for such components; and (iii) were published in 2016 or later. Records were excluded if they were duplicates, purely incremental reprints, concerned non-ferromagnetic materials only, or lacked sufficient methodological or bibliographic detail to classify. This review centers on machine vision and MFL; other NDT modalities (laser/3D profilometry, eddy-current arrays, ACFM, thermography) are considered as fusion partners or methodological precedents. For fusion-evidence classification, direct camera–MFL studies require independently acquired optical and MFL measurements of the same physical defects, and signal-derived images retain their underlying sensing modality. Deduplication by DOI and normalized title removed 308 records, leaving 2166 unique records for title/abstract screening, of which 1968 were excluded as off-topic. The remaining 198 records were assessed in full text, and 76 were excluded: 72 were off-scope or lacked a described method and evaluation (e.g., abstract-only records and product notes), and 4 had incomplete bibliographic records. The experiment-reporting requirement applied to records claiming original research. Review and survey articles, and works on non-ferromagnetic objects or cross-domain methods that offer transferable fusion, registration, or robotic-carrier methods, were retained through a separate background and positioning channel (categories A and B below). This step yielded 122 included works; the four surveys added on 12 October 2026 bring the final corpus to 126. The retained corpus comprises 35 works on vision-based detection, 43 on MFL and other electromagnetic methods, 31 on multimodal fusion, and 17 on robotic acquisition platforms; 95 are English-language and 31 are Chinese-language publications; and by document type, 104 are original research articles and 22 are reviews or surveys. Of the 126 works, 111 address steel or other ferromagnetic components directly and form the core corpus, while 15 serve as adjacent-domain evidence in two categories: (A) nine works whose inspection objects are entirely non-ferromagnetic: food and plant monitoring [28,29], tire and building inspection [30,31], composite and friction-stir-weld testing [32,33,34], and concrete-structure robots with visual or hammering inspection [35,36]; and (B) six cross-domain methodological precedents: cross-material fatigue prediction [37], general incomplete-modality learning [38], multimodal ultrasonic sensor fusion [39], laser-thermography fusion [40], general multimodal perception [41], and broad NDT image fusion [15]. No formal quality-appraisal tool was used; the inclusion criteria served as a minimum reporting standard. Corpus statistics describe all 126 works, and the reference list comprises these 126 works plus one standard (API 1163) cited for context. Claims about steel image–MFL inspection draw on the relevant core studies; Table S1 distinguishes direct pairing evidence from adjacent examples.

### 2.2. Statistical Landscape

**Figure 4:**
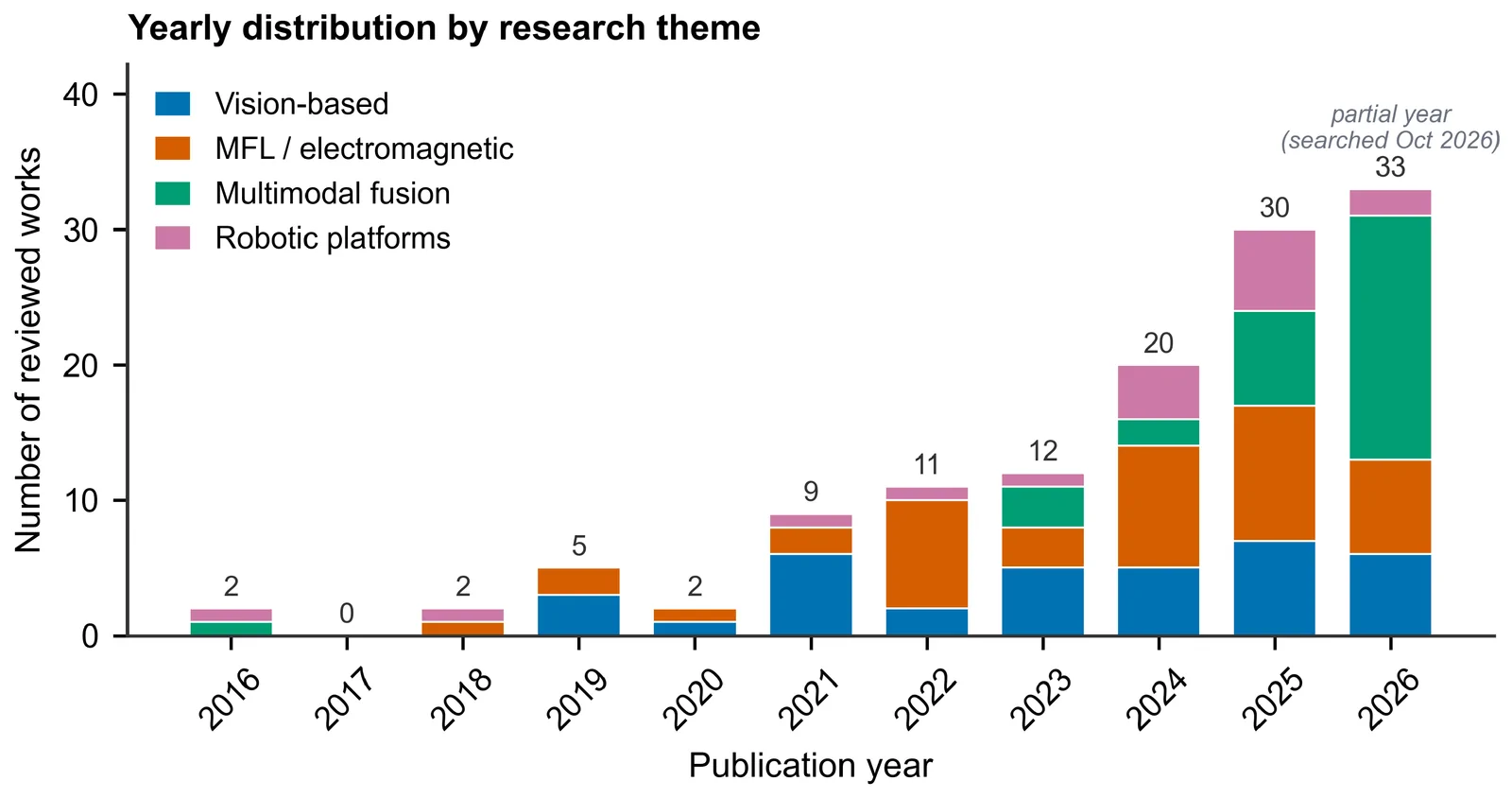
Yearly distribution of the 126 reviewed works by research theme. The 2026 bar is a partial year (searched October 2026). The distribution of publication venues and journal quartiles is given in Figure S2 of the Supplementary Materials.

The statistical profile of the corpus is shown in Figure 4. The annual output has grown markedly since 2021, and about 91% of the reviewed works were published in 2021 or later (115 of 126). Fusion works in the corpus rose from 3 in 2023 to 7 in 2025, with 18 in the partial year 2026 (Figure 4). The distribution of publication venues and journal quartiles is reported in Figure S2 of the Supplementary Materials.

## 3. Vision-Based Surface Defect Detection

Machine vision is the most intuitive modality for steel surface inspection: industrial cameras capture surface morphology, and image processing or deep learning algorithms localize, segment, and classify defects. Research in this theme can be organized into image acquisition and preprocessing, classification networks, detection networks, segmentation networks, and learning under data-limited regimes.

### 3.1. Image Acquisition and Preprocessing

The quality of visual inspection is bounded by imaging conditions. Steel pipe interiors and large curved surfaces present non-uniform illumination, reflective glare, shadowing at weld seams, blur induced by robot vibration, and occlusion by welding residues or rust [1,6]. Hardware-side solutions include structured lighting, photometric stereo, and multi-camera or depth-camera arrays; for example, photometric-stereo vision has been used to reveal shallow grinding defects on rail weld surfaces [42], and depth cameras have been exploited to fuse grayscale and depth features for steel pipe surface inspection [43]. On the algorithm side, illumination correction, deblurring, and contrast enhancement are routinely applied before detection; several recent studies embed enhancement modules directly into the detection network so that restoration and recognition are optimized jointly [44,45]. These preprocessing and joint-enhancement strategies are particularly relevant for robot-carried cameras, where illumination geometry changes continuously with the robot pose.

### 3.2. Classification Networks

Classification networks determine whether an image patch contains a defect and, if so, of what type. Early deep-learning pipelines transferred general-purpose backbones (AlexNet, VGG, GoogLeNet, ResNet, DenseNet, and lightweight variants such as MobileNet and ShuffleNet) to steel surface datasets [1,3]. Fu et al. proposed a fast and robust classification approach that remains accurate under non-uniform illumination [46], and Di et al. introduced a semi-supervised classification method that exploits the large proportion of unlabeled steel surface images [47]. Deep convolutional networks have been systematically validated for automatic detection and classification of steel surface defects [48], and dedicated algorithms targeting the NEU-DET benchmark continue to refine accuracy–efficiency trade-offs [49]; smart-monitoring-oriented inspection approaches further connect classification models with industrial IoT pipelines [50]. Comparative studies have shown that classification networks are effective for screening but cannot provide defect location or extent, which limits their standalone value in industrial grading tasks [2,10].

### 3.3. Detection Networks

Object detection networks localize defects with bounding boxes and assign categories, and they currently dominate engineering deployments. Single-stage detectors of the YOLO and SSD families provide the best accuracy–speed trade-off and are the mainstream choice for online inspection, while two-stage detectors such as Faster R-CNN remain accuracy references [2,4,10]. Reported improvements cluster around four recurring targets: small and low-contrast defects, addressed by multi-scale feature fusion, attention mechanisms, and transformer modules [51,52,53,54]; deployment cost, addressed by lightweight and edge-oriented designs [44,45,55,56]; domain-specific imagery, including weld X-rays, stainless-steel pipe surfaces, steel members, and pressure-pipeline welds [57,58,59,60]; and industrial validation beyond benchmark scores [61,62]. The Chinese-language literature follows the same engineering trend, with improved YOLO variants for steel surfaces, weld real-time detection, and grayscale–depth feature fusion [43,63,64].

### 3.4. Segmentation Networks

When defect shape, area, and boundary are required for quantitative evaluation, segmentation networks are preferred. Semantic segmentation assigns a category to every pixel (FCN, U-Net, DeepLab), whereas instance segmentation additionally separates individual defects (Mask R-CNN, PANet) [5,10]. Transfer-learning variants of U-Net have demonstrated accurate steel surface segmentation with limited training data, and improved Res-UNet designs with Dice-type losses alleviate the foreground–background imbalance that is typical of defect imagery [4]. Segmentation outputs also serve as precise regions of interest (ROIs) for subsequent quantitative measurement and for guiding other modalities, a role examined in Section 5.

### 3.5. Learning Under Data-Limited Regimes

Severe defects are rare by nature, so labeled samples are scarce and class-imbalanced. Few-shot, semi-supervised, and unsupervised approaches have therefore attracted increasing attention. Semi-supervised classification of steel surfaces [47], adaptive graph neural networks with semi-supervised multimodal fusion for few-shot strip steel classification [65], and unsupervised reverse-distillation learning for pipe weld defect detection [66] explore reduced-label learning for surface and weld inspection. Surveys of real-time inspection [11] and of automated visual detection on flat steel [2] consistently identify data scarcity, domain shift between production lines, and the precision–latency trade-off as the three dominant obstacles to deployment. Ultrasonic-image-based weld recognition with CNNs [67] further illustrates how such models are being embedded into practical inspection workflows, while intelligent detection and grading of welded joints in pressure equipment that incorporates industry standards points toward certification-oriented deployment [68]. Broader overviews of machine-vision methods for metal surfaces and steel plates are given in [69,70,71]. Table 2 lists ten representative vision-based studies published between 2019 and 2026. The entries were selected to cover classification, detection, segmentation, and few-shot learning and to include studies that report results on a named dataset. The “Key technique” column gives the main methodological contribution of each study, and the “Critical remarks” column records its evaluation protocol and principal limitation. Most quantitative results in the table come from flat-steel benchmarks (NEU-DET, GC10-DET) or single-site in-house datasets, so scores obtained on different datasets or under different IoU protocols should be read separately; independent test sets and validation beyond benchmark data are rarely reported.

### 3.6. Reported Performance and Remaining Gaps

Reported NEU-DET results use different IoU protocols: MSFT-YOLO reports 75.2% mAP@[.5:.95] [52], whereas WSS-YOLO and DSAT report 82–83% mAP@0.5 [44,54]. These values do not establish a temporal improvement trend. For the same model and dataset, DSAT reports 83.14% mAP@0.5 and 47.37% mAP@[.5:.95] on NEU-DET [54], showing that precise localization of small and low-contrast defects (crazing and rolled-in scale are consistently the hardest categories) remains far from solved even when coarse detection looks strong. The same study reports 67.27% mAP@0.5 and 34.09% mAP@[.5:.95] on GC10-DET with separately trained models [54]; the two sets of scores describe different dataset-specific tasks, not cross-domain transfer.

Works evaluated on in-house visual-inspection datasets (a weld X-ray set [57], a GMAW weld image set [53], and a stainless-steel tube surface-crack set [58]) report markedly higher numbers (92.6%, 95.3%, and 97.9% mAP@0.5, respectively). These scores come from single-site datasets with different imaging physics and defect taxonomies. Interpretation of high benchmark scores also depends on split independence and label quality: NEU-DET comprises 1800 grayscale images in six classes, and image-level random splits risk near-duplicate leakage between training and test partitions (see Section 4.4). Encouragingly, some studies go beyond benchmark scores: YOLO-MSAPF reduced the image-level miss rate from 21.47% to 1.68% while cutting parameters by 30.1%, and validated screening performance in an industrial environment [53]; WD-YOLO was deployed on a production line where its 98 FPS inference outpaced the X-ray imaging rate [57]. Such miss-rate and throughput evidence is more meaningful for acceptance decisions than incremental benchmark gains and merits systematic reporting in future vision studies.

Most reviewed visual results come from flat manufacturing-stage surfaces or weld radiographs, under controlled imaging geometry. Curvature, robot pose, and in-service damage change the inspection task [6,17]: surface curvature induces perspective distortion and illumination gradients that flat-surface models are not trained on, and robot-carried cameras add pose-dependent blur and shadowing. Weld radiographs represent volumetric projections rather than optical surface images, and their defect classes (porosity, lack of fusion) differ from surface-breaking defects. The STF study [58] does not report the steel grade or magnetic properties of its specimens. Transfer to curved, in-service steel and compatibility with MFL therefore remain to be evaluated.

## 4. MFL-Based Defect Detection

MFL testing magnetizes a ferromagnetic component toward saturation; at defects, the local magnetic reluctance changes and flux leaks into the air, where it is captured by Hall elements or induction coils. MFL is non-contact, couplant-free, fast, and sensitive to both surface and near-surface defects, which has made it the dominant in-line inspection technique for oil and gas pipelines [9,12,72]. Research in this theme covers probe and system design, signal processing, deep-learning-based interpretation, and defect quantification.

### 4.1. Probes, Magnetizers, and Inspection Systems

Signal quality is largely determined by the sensing front end. Engineering studies have optimized excitation coil configurations, magnetic shielding and flux-guiding structures, and tri-axial sensor arrangements [9]. Ege and Coramik designed a complete MFL measurement system for pipeline inspection [72]; Ding et al. developed an MFL system for quantitative identification of broken-wire defects in bridge cables [73]; and Long et al. proposed an ultra-high-definition MFL detection topology for crack quantification [74]. For curved surfaces and micro-defects, flexible eddy-current/MFL array sensors and dual-square-winding probes have improved sensitivity and conformability, and review-level analyses of eddy-current probe design summarize transferable principles for sensor optimization [7,8]. High-speed inspection introduces additional distortions; Ren et al. quantified velocity-induced deformation in MFL signals of weld defects [75], an effect that must be compensated when sensors are carried by moving robots. Experiment–simulation co-analysis of MFL testing for steel plates with corrosion defects provides validated signal references for sensor and algorithm design [76].

### 4.2. Signal Processing and Traditional Machine Learning

Classical MFL interpretation chains comprise denoising (wavelet thresholding, empirical mode decomposition, adaptive filtering), feature extraction (time-domain amplitude, zero-crossing time, frequency and time–frequency coefficients), and classifiers such as support vector machines, random forests, and shallow neural networks [9,12]. Wavelet-transform-based fusion has been applied to edge detection of MFL anomalies [77], and Kriging interpolation has been used for fast defect localization in large-scale pipeline data [78]. These methods are interpretable and data-frugal, but their handcrafted features generalize poorly across pipe diameters, magnetization levels, and lift-off variations [13].

### 4.3. Deep Learning for MFL Interpretation

Deep learning has reshaped MFL data analysis in three ways [13]. First, one-dimensional signals are processed directly by temporal networks: CNN and LSTM stacks learn discriminative patterns from raw tri-axial waveforms, and noise-robust frameworks improve weld-defect detection under strong interference [79]. Second, MFL data are transformed into images so that mature vision models can be reused; cascaded deep models combining Swin-Transformer-backbone detectors with quantification heads have been proposed for pipeline defect detection and sizing, a cascaded YOLOv11 detector coupled with a physics-informed network performs MFL defect detection and size estimation in sequence [80], lightweight online weld-defect detection has been demonstrated on real inspection systems [81], and cascade attention networks improve defect recognition under complex magnetic backgrounds [82]. In the Chinese literature, YOLOv8-based intelligent recognition of pipeline MFL defects [83] and small-defect detection networks with shallow-feature suppression [84] follow the same direction. Third, physics-informed learning embeds electromagnetic priors into neural networks: physics-informed neural networks have been used for MFL defect size estimation [85], machine-learning-based dipole modeling reconstructs three-dimensional MFL fields of outer-surface cracks [86], and physics-guided evolutionary deep learning addresses dynamic defect inversion under stress–hydrogen coupling interference in hydrogen-blended pipelines [87,88]. Semi-supervised learning on MFL signals further reduces labeling costs [89], generative approaches are emerging for data augmentation: diffusion models have been used to synthesize realistic MFL signals that improve downstream defect-recognition accuracy [90], and complex-valued CNNs reconstruct carbon-steel defects directly from eddy-current signals [91]. Data-driven models embedding time-frequency representations have also detected small curved metallic cracks from flux leakage [92], and dual-driven AI schemes have been explored for eddy-current array tomography of surface defects [93]. Related progress in adjacent electromagnetic modalities, such as AC field measurement with tri-axial information fusion for defect quantification [94], pulsed eddy-current thermography crack detection via deep transfer learning [95], and electromagnetic tomography imaging with edge-guided segmentation [96], enriches the methodological toolbox available to MFL interpretation.

### 4.4. Defect Quantification, Inversion, and Datasets

For integrity assessment, detection must be followed by quantification: estimating defect depth, width, and length from MFL measurements. CNN-based inverse models predicting corrosion size and location [97], classification-and-regression networks for pinhole corrosion [98], tri-axial fusion inversion of in-line inspection data [99], combined-diagnostics sizing validated against ultrasonic references [100], and joint quantification frameworks that integrate magnetic-flux measurements with an image-processing branch [101] demonstrate steady progress. A persistent bottleneck has been the lack of public benchmarks; the release of PipeMFL-240K, a large-scale dataset and benchmark for object detection in pipeline MFL data, marks an important step toward reproducible comparison [102]. Reviews of deep learning for MFL evaluation of oil and gas pipelines [13] and of MFL NDT more broadly [9] both emphasize that paired, well-annotated industrial datasets remain scarcer than algorithms. Ultrasonic weld recognition studies [103] and laser-cladding defect recognition with interpretable deep networks [104] complement the electromagnetic evidence and are natural candidates for fusion, as discussed next.

Table 3 lists 14 representative MFL studies published between 2018 and 2026, selected to span system and probe design, signal analysis, deep-learning interpretation, quantification, data augmentation, and benchmarking. The “Focus” column identifies the stage of the interpretation chain addressed by each study, and the “Critical remarks” column records the data source and the main limitation. Most quantification entries rest on finite-element or dipole-model simulations and laboratory specimens; evaluation on field data with physical sizing references is rare (Section 4.5).

Table 4 collects the seven datasets behind the quantitative comparisons in this review: three public vision benchmarks, the PipeMFL-240K benchmark, and three in-house sets that produced the highest reported vision scores. For each dataset, the table gives the modality, scale, class structure, and availability. The three in-house sets are either not public or available only on request, and PipeMFL-240K is the only large-scale public MFL dataset; no public dataset of spatially registered image–MFL pairs was identified.

### 4.5. Reported Performance and the Quantification Gap

MFL benchmark results reveal substantial remaining challenges. On PipeMFL-240K, the large-scale public MFL image benchmark (Table 4), the best of the 22 benchmarked detectors, YOLOv5-l and YOLOv8-x-world-v2, reach mAP@0.5 = 0.498 on the test set [102]. The best structural-component categories (valves, sleeves) exceed AP50 0.8, whereas damage categories such as metal loss and girth-weld anomalies remain below AP50 0.3 [102]. The main test split measures within-pipeline performance, while the separate zero-shot set tests an unseen pipeline [102]. Because the benchmark derives from in-service inspection data, these results address a different dataset and task from the visual studies in Section 3.6. Data volume is a first-order constraint: with 1/16 of the training data, YOLOv8-m drops from 0.450 to 0.169 mAP@0.5, while RF-DETR-Base degrades considerably less [102].

For quantification, the step that turns detection into integrity decisions, most reviewed sizing results are based on simulation or laboratory measurements. CNN inverse models trained on finite-element MFL signals with injected noise achieve high sizing accuracy on simulated corrosion defects [97], physics-informed networks improve both accuracy and physical consistency over purely data-driven regressors on dipole-model data [85], cascaded detector–regressor pipelines now combine learned detection with physics-informed sizing [80], and spatial-integration quantification achieves depth and width errors within 0.7 mm and 0.1 mm, respectively, for high-aspect-ratio cracks in physical experiments [74]. Few reviewed studies report sizing error against excavated or ultrasonic references [100], limiting assessment under field variability.

## 5. Multimodal Fusion Strategies and Cross-Study Synthesis

Single modalities capture complementary aspects of a defect: vision excels at morphology and localization, while MFL and related electromagnetic signals penetrate the surface and respond to hidden discontinuities. Fusion research aims to convert this complementarity into measurable gains in detection performance [14,15]. This section distinguishes direct camera–MFL studies from adjacent NDT pairings and organizes the evidence by fusion level and comparison design.

### 5.1. Fusion Levels

**Data-level fusion** aligns raw or minimally processed data from multiple sensors in a common spatial or temporal reference and feeds the combined representation to a single model. Multispectral registration frameworks with coarse-to-fine alignment demonstrate that registration quality largely determines the achievable fusion gain [15,40]. For image–MFL systems, data-level fusion requires accurate mapping between camera pixels and MFL sampling positions, which is non-trivial when the two sensors are mounted on different devices or operated at different times.

**Feature-level fusion** extracts modality-specific embeddings and merges them through concatenation, weighting, gating, or attention. Deep multimodal fusion of spectral and visual data has improved laser-welding defect classification [105], and generative–recurrent hybrids (WGAN-GP with Bi-GRU) have fused multiple sensing channels for damage identification [106]. Fusion of visual and NDT features outperformed a single YOLOv8x baseline for construction quality inspection [107]. Multimodal fusion combined with transfer learning has even enabled cross-material life prediction of defect-dominated fatigue [37], and infrared thermography fused with air-coupled ultrasound improves delamination detection in composites [32].

**Decision-level fusion** keeps independent per-modality pipelines and combines their outputs through voting, weighted logic, Bayesian rules, or Dempster–Shafer evidence theory. A well-documented NDT example is the scattered decision-level fusion of eddy-current, laser-active thermography, and MFL inspections of a curved steel bearing shell with artificial grooves, where kernel-density aggregation of per-sensor detections improved detection reliability over any single sensor, and leave-one-sensor-out experiments quantified each sensor’s contribution [14]. Object-level registration of infrared thermography with visual inspection, combined through probabilistic fusion, has been validated for pre-operational inspection of large-diameter pipelines [23]. Physics-guided evidential frameworks quantify and propagate uncertainty, which is valuable for small-sample weld defect detection [108]. Decision-level fusion can accommodate asynchronous inputs and sensor dropout when the fusion rule includes explicit missing-modality handling, at the cost of weaker exploitation of low-level cross-modal correlations [15]. Table 5 compares the three fusion levels.

### 5.2. Fusion of Electromagnetic and Acoustic Modalities

Before vision–electromagnetic fusion became feasible, the NDT community accumulated substantial experience in fusing MFL/ECT with ultrasonic modalities. Combined UT–MFL or EMAT–ECT probes improve the detection of both surface and internal defects, and physical coupling fusion sensing of MFL and EMAT enables synchronous inspection of surface and internal defects in ferromagnetic steels [16]. Ultrasonic guided-wave fusion networks have been explored for anchor-bolt crack detection [110], and deep-learning interpretation of infrared thermography has proven effective for friction-stir-weld defect detection [34]. These works establish an important lesson for image–MFL fusion: probe-level co-design and signal separability should be considered before algorithmic fusion [15,16].

### 5.3. Vision–Electromagnetic Fusion and Cross-Modal Guidance

Vision–electromagnetic fusion is the youngest but fastest-growing branch. Domain-knowledge-guided networks have been proposed for intelligent girth-weld defect detection in pipelines [111]; multimodal deep learning has been demonstrated for stainless-steel pipe manufacturing lines [112]; and non-invasive coating defect detection combines visual assessment with multimodal validation [113]. Magneto-optical imaging combined with deep feature extraction has improved defect classification [114]; the fusion there occurs within one imaging-physics pipeline. In adjacent monitoring domains, image–vibration fusion has improved condition assessment of transportation infrastructure [115].

Gao et al. [109] report a serial camera–MFL workflow in which visual recognition based on deep transfer metric learning is followed by sizing from Gramian-angular-field-encoded MFL signals. It provides a serial baseline for evaluating registered fusion. A further candidate [101] integrates magnetic-flux and image data with a dedicated image-processing branch; the accessible record does not establish whether the image branch uses independently acquired optical data, so its camera–MFL status remains unresolved.

Beyond simple fusion, *bidirectional cross-modal guidance* is proposed here as a testable direction for registered image–MFL systems. Visual defect maps could guide MFL ROI selection and localized inversion, while MFL measurements could help distinguish defects from visual artefacts. MFL does not require optical access, but coatings can alter lift-off. Magnetization, velocity, and geometric transitions also affect the response. Visual ROIs should therefore be treated as uncertain priors, and cross-modal reconstruction should be restricted to geometrical quantities supported by measurements. Related approaches appear in knowledge-fused weld radiograph interpretation [116] and embodied intelligent NDT systems [41]. Within ferromagnetic infrastructure, lightweight multi-scale fusion detectors for rail surfaces show that fused representations can meet real-time budgets [117].

### 5.4. Transformer-Based Fusion Architectures

Transformers are widely used as fusion backbones because self- and cross-attention can model long-range dependencies between heterogeneous token sequences. In the reviewed corpus, transformer backbones appear both inside single modalities (e.g., Swin-backbone detectors for MFL images, dynamic sparse attention for steel surfaces [54]) and as explicit fusion modules that learn modality weights adaptively. Encoder-based fusion with modality-specific encoders, a fusion encoder, and task heads is illustrated by spectral–visual laser-welding monitoring [105]. Single-modality multi-scale networks such as MIF-YOLOv8 on weld radiographs [111] are classified with the vision evidence. Open questions include how to inject physical priors (magnetization state, lift-off, velocity) as tokens or constraints, and how to keep fused models interpretable enough for safety-critical grading [41,108].

### 5.5. Hybrid Fusion, Missing Modalities, and Fusion-Quality Metrics

The three canonical fusion levels are increasingly combined rather than used in isolation. Hybrid pipelines cascade data-level alignment with feature-level interaction, inheriting the information retention of early fusion and the flexibility of intermediate fusion. For image–MFL acquisition by different devices at different times, however, a more pressing issue is *missing-modality robustness*: illumination failures, occlusions, or skipped MFL passes mean that one modality is intermittently absent at inference time. Cross-modal knowledge distillation for incomplete multimodal data, which transfers information from a modality-rich teacher to a modality-poor student, has been formalized in general multimodal learning [38] and provides a candidate approach to single-modality fallback in asynchronous inspection. Fusion-quality evaluation should report registration tolerance, performance under modality removal or corruption, and uncertainty calibration; Table 6 lists candidate evaluation metrics and their measurement status in the reviewed corpus. Entries reading “not reported” are search findings: no study within the search scope reports that quantity for the stated pairing.

### 5.6. Quantified Evidence of Fusion Gains

Table 7 separates controlled comparisons, system-level changes, capability demonstrations, and observed failures; these designs support different conclusions about added-modality benefit.

**(i) Same-study controlled comparisons** evaluate fused and single-modality variants under matched conditions [14,65,105]; the reviewed instances concern image–text, spectral–visual, and ECT–thermography–MFL pairings. The 5.37-percentage-point few-shot estimate of Kong et al. [65] is excluded because the reported comparator and task definition do not support attribution to the text modality (Table 7, note a).

**(ii) System-level before/after changes** evaluate complete pipelines rather than isolated modality contributions [65], and **(iii) capability demonstrations** report the detectable defect range [16]; neither design isolates the contribution of the added modality. The accessible record for He et al. [108] supports probabilistic fusion evidence; a matched fused-versus-single-modality comparison remains unconfirmed.

**(iv) Fusion failures also matter**: strict agreement rules can reject true indications detected by only one sensor [14]. Image–MFL evaluation should therefore test inter-sensor conflict and modality removal alongside detection and sizing performance. The serial camera–MFL workflow is discussed in Section 5.3. Within the search scope, no study has shown that spatially registered camera–MFL fusion outperforms matched single-modality baselines, and no public registered benchmark was identified.

### 5.7. Cross-Study Synthesis and the Limits of Comparison

This subsection synthesizes the quantitative results reported by representative works. Studies were selected for Table 8 if they report evaluation results on a named dataset and together span the 2018–2026 period and both modalities.

**Figure 5:**
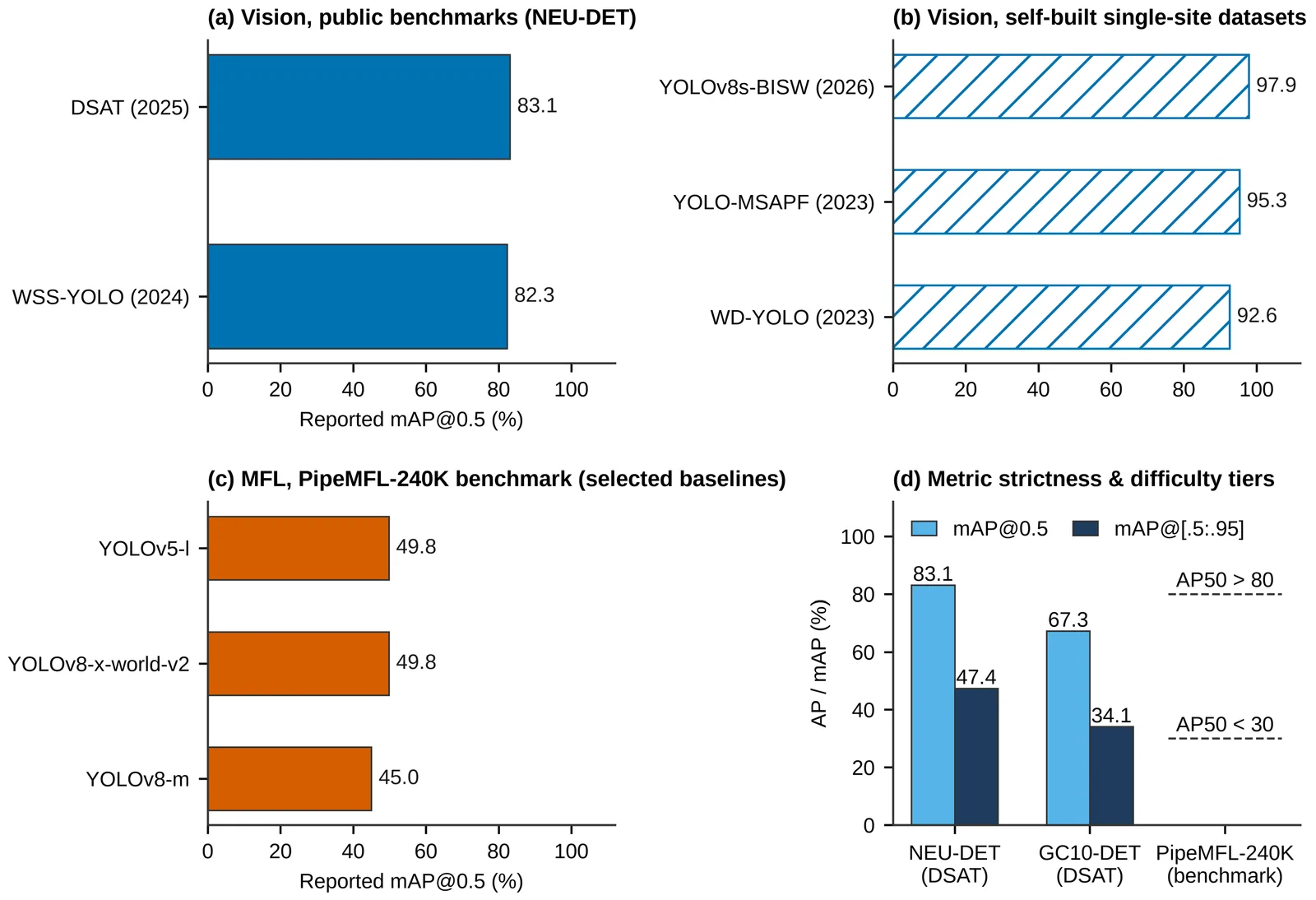
Reported performance across tasks. (**a**) Vision detectors on public benchmarks [44,54]; (**b**) vision detectors on self-built, single-site datasets [53,57,58] (hatched); (**c**) selected detector baselines on the PipeMFL-240K MFL benchmark [102]; (**d**) within-study effect of metric strictness (DSAT on NEU-DET and GC10-DET [54]) and the PipeMFL-240K category-difficulty bands [102]. Panels use task-specific datasets and metrics and do not support cross-panel rankings; panel (**a**) includes mAP@0.5 results only. In panel (**d**), PipeMFL-240K reports category-level AP50 only as bounds, shown as dashed reference lines: the best component classes exceed 80 and the damage classes remain below 30.

Differences in dataset (1800 vs. 240,320 images), evaluation split, IoU protocol (mAP@0.5 vs. mAP@[.5:.95] can differ by a factor of 1.7 on the same model [54]), hardware, and defect taxonomy limit direct comparison of the values in Table 8. Standardized evaluation (common datasets, splits, metrics, and reporting of miss rate and throughput alongside mAP) is a prerequisite for cumulative progress [15,102]. Figure 5 visualizes the two task-specific observations: vision detectors report high mAP@0.5 on curated benchmarks, with substantially lower strict-IoU scores within the same studies, while MFL detectors leave substantial room for improvement on the large-scale public MFL benchmark.

## 6. Robotic Acquisition, Open Challenges, and Future Directions

Field-deployable dual-modality inspection depends on carriers that bring sensors to the surface reliably. Two families dominate: pipeline in-line inspection robots and magnetic-adhesion wall-climbing robots. This section examines both carriers and their implications for dual-modality acquisition, then turns to the open challenges and future directions that follow from the evidence reviewed.

### 6.1. Pipeline In-Line Inspection Robots

PIG-style carriers transport MFL, caliper, and increasingly vision payloads through pressurized pipelines over tens of kilometers. Recent systems integrate phased-array ultrasonic units on large-diameter gas pipelines [118], and automated box-data matching has improved the alignment of multi-modal MFL inspection runs [119]. Because of their cost and diameter constraints, in-line robots are primarily used for transmission pipelines; distribution pipes, tanks, and structural members require more agile carriers.

### 6.2. Magnetic-Adhesion Wall-Climbing Robots

Wall-climbing robots adhere to ferromagnetic surfaces through permanent magnets or electromagnetic units and carry cameras, lighting, and NDT payloads to vertical and overhead regions. Surveys of magnetic wall-climbing robots [17,18] and of smart corrosion-inspection robots with IoT integration [20] document rapid progress in adhesion mechanisms, locomotion, and autonomy. Application-driven designs include climbing robots for steel bridge inspection with visual and three-dimensional mapping [19,120], machine-vision-based crawlers for automatic weld-seam tracking [21], wheeled permanent-magnet robots for weld defect detection on hydraulic steel gates [121], wall-climbing robots with manipulators for pressure-vessel weld inspection [122], heavy-load platforms for bridge concrete inspection [36], propeller-type robots for visual and hammering inspection of concrete [35], and magnetic-particle testing robots for ship welds and cylindrical tanks [22,123]. Weld-dressing robots demonstrate that climbing platforms can also support repair operations [124], and optimized laser weld-image classification on robot-acquired imagery closes the loop between acquisition and recognition [125]. Reviews of inspection robots for confined special equipment summarize domain-specific constraints on payload, adhesion margin, and explosion protection [126].

### 6.3. Implications for Dual-Modality Acquisition

For image–MFL dual-modality systems, the platform determines what fusion is feasible. Co-mounted and separate-pass acquisition impose different payload, interference, and registration requirements. A co-mount must account for sensor spacing, magnetizer layout, and a shared calibration frame, and co-mount design should test whether camera electronics and illumination require shielding from the magnetizer. Separate passes require component/defect correspondence, spatial alignment, and control of the measurement state. The fusion level should be selected against the registration tolerance required by the intended task; this tolerance can be expressed as a registration error budget, i.e., the residual misalignment between image pixels and MFL samples that each fusion level can tolerate. The budget links mechanical layout choices to the fusion-quality metrics of Section 5.5. Potential co-mounting effects include adhesion-induced pre-magnetization, sensor saturation near the adhesion magnets, and lift-off jitter from wheel contact and suspension compliance. Crawler speeds (typically 0.05–0.3 m/s [17,18]) lie far below in-line inspection tool speeds (about 1–5 m/s [72,118]), so robot-borne MFL interpretation needs evaluation across the intended velocity and lift-off conditions. Magnet spacing, surface curvature, and pose estimation should be assessed for their effects on registration and sizing. Figure S1 in the Supplementary Materials illustrates the authors’ planned testbed for such registration experiments; curved specimens with controlled artificial defects provide a practical basis for validating registration and fusion pipelines [21,22,122]. Table 9 relates acquisition choices to inspection conditions and supporting evidence.

### 6.4. Data and Registration

Public datasets exist separately for vision and, recently, for MFL [102]. Paired datasets should document sensor geometry, acquisition timing, surface state, defect identity, and reference dimensions. Spatial fusion requires a shared coordinate frame; serial workflows require component or defect correspondence. Few-shot, semi-supervised, and unsupervised learning [47,65,66,89] and cross-modal synthesis (e.g., simulating MFL responses from visual defect maps through electromagnetic simulation) are promising mitigations for data scarcity; odometry, fiducial markers, and surface reconstruction must establish a unified coordinate frame, and fusion algorithms should explicitly tolerate registration uncertainty [14,119]. Standardized collection protocols on specimens with certified artificial defects would accelerate both.

### 6.5. Physics and Sizing Reliability

Cracks, scratches, pits, inclusions, laps, and weld imperfections differ drastically in scale, texture, and magnetic signature, and compound defects interact non-linearly [1,13]; a unified representation spanning morphological and electromagnetic signatures remains open. Grading decisions ultimately depend on defect dimensions, so quantification errors propagate directly into safety margins. Physics-informed inversion [85,86,87] and cross-validation against complementary modalities [100] are promising, but field-validated sizing errors remain scarce (Section 4.5); sizing studies should report errors against physical references under the intended field conditions. API 1163 provides an in-line inspection qualification context [127]; its applicability to crawler-based inspection requires separate justification. Uncertainty quantification should accompany every quantitative output [108].

### 6.6. Field Conditions and Standardization

Pipe interiors and curved structures combine narrow space, uneven illumination, weld spatter, rust, and occlusion, under which vision models degrade sharply [6,11], while lift-off variation, velocity effects, and magnetic noise contaminate MFL signals [9,75]; field pipelines need illumination correction, compensation for MFL lift-off and velocity effects, and evaluation under domain shift. Online inspection additionally requires models that run at robot speed on embedded hardware: lightweight detectors [44,55,56] and online MFL weld-defect detection [81] reconcile accuracy with latency; for fused dual-modality models, compression, distillation, and hardware-aware design are candidate mitigations. Finally, paired-data studies should use common defect definitions and report splits, detection, localization, and sizing metrics consistently [15,102].

### 6.7. Future Directions

Four research directions address these constraints. (i) Bidirectional cross-modal guidance, in which visual priors guide MFL denoising and localized inversion while MFL evidence suppresses visual false alarms under occlusion, should be tested on registered pairs against the evaluation targets of Table 6. (ii) Transformer-based fusion with explicit physical tokens and constraints (magnetization state, lift-off, velocity) can bridge data-driven learning and electromagnetic forward models [85,105]. (iii) The joint design of sensor placement and robot motion should optimize the mechanical and magnetic layout against the registration error budget and motion constraints [17,21]. (iv) Foundation-model approaches that pool knowledge across inspection tasks are a longer-term option [37,41]. All candidate architectures should be evaluated against strong single-modality and serial baselines using the targets in Table 6.

## 7. Conclusions

This review has examined how far published evidence supports image–MFL dual-modality inspection for steel pipes and curved steel surfaces across 126 works (2016–2026), positioned against fifteen prior surveys. Vision detectors reach 82–83% mAP@0.5 on public benchmarks and up to 97.9% on self-built datasets, but within-study strict-IoU scores drop to about 47% [54] and most evidence comes from flat, manufacturing-stage material. On the large-scale public PipeMFL-240K benchmark, the best detectors reach mAP@0.5 = 0.498, with damage categories far below structural components, and quantification remains validated mostly on synthetic rather than field data. Within the search scope, no study has shown that spatially registered image–MFL fusion outperforms matched single-modality baselines. Paired data, cross-device registration, field-validated sizing, and consistent evaluation are therefore the immediate priorities, particularly paired measurements acquired by robotic carriers under field conditions. 

## Figures and Tables

**Table 1:** Positioning of this review against existing surveys in adjacent areas. “partial” marks topics present but not analyzed as a primary subject. The 2025–2026 entries [24,25,26,27] were added after the October 2026 query extension and a re-check of title-level exclusions; for [24], coverage is assessed from the publisher’s abstract and bibliography.

Review	Year	Scope	Vision	MFL	Image–MFL	Robotic
					Fusion	Carriers
Luo et al. [2]	2020	Visual defect detection for flat steel	✔	–	–	–
Chen et al. [3]	2021	Surface defect detection, industrial products	✔	–	–	–
Bhatt et al. [5]	2021	Deep learning for image-based surface defects	✔	–	–	–
Zheng et al. [4]	2021	Deep-learning surface inspection, industrial products	✔	–	–	–
Usamentiaga et al. [10]	2022	Detection and segmentation review, metal surfaces	✔	–	–	–
Wen et al. [1]	2023	Steel surface defect recognition	✔	–	–	–
Liu et al. [11]	2023	Real-time deep-learning surface inspection	✔	–	–	–
Tang et al. [6]	2023	Machine-vision detection for steel products	✔	–	–	–
Feng et al. [9]	2022	MFL nondestructive testing	–	✔	–	–
Huang et al. [13]	2023	Deep learning for pipeline MFL evaluation	–	✔	–	–
Qiu et al. [24]	2026	Pipeline MFL: physical models, AI, multi-modal sensing	partial	✔	partial	partial
Ma et al. [25]	2025	Pipeline in-line inspection technologies	partial	✔	–	partial
Khalid et al. [26]	2026	Pipeline damage detection and localization	partial	partial	partial	–
Xu et al. [27]	2026	Pipeline robots in oil and gas	partial	partial	–	✔
Torbali et al. [15]	2023	NDT image fusion across modalities	✔	partial	partial	–
**This review**	2026	Image–MFL inspection: evidence, fusion strategies, acquisition	✔	✔	✔	✔

**Table 2:** Representative vision-based steel surface defect detection works (2019–2026).

Study	Year	Category	Key Technique/Contribution	Critical Remarks
He et al. [51]	2020	Detection	End-to-end CNN fusing multiple hierarchical features	High accuracy; limited real-time validation
Di et al. [47]	2019	Classification	Semi-supervised learning with unlabeled surface images	Classification only; no defect localization
Guo et al. [52]	2022	Detection	MSFT-YOLO: transformer module embedded in YOLOv5	NEU-DET; 75.2% mAP@[.5:.95]
Wang et al. [53]	2023	Detection	YOLO-MSAPF: multiscale alignment fusion with parallel filtering	Single-site weld dataset; cross-site generalization not reported
Lu et al. [44]	2024	Detection	WSS-YOLO: trade-off between detection accuracy and computational cost	Reports 82.3% (NEU-DET) and 72.0% (GC10-DET) mAP@0.5; scores strongly dataset-dependent
Wu et al. [54]	2025	Detection	DSAT: dynamic sparse attention transformer	Strict-IoU protocol costs 35.77 percentage points on NEU-DET (47.37% vs. 83.14% mAP@0.5)
Wang et al. [45]	2026	Detection	MSAF-YOLO: efficient multi-scale attention fusion network	Deployment-oriented; external validation beyond benchmark data not reported
Kong et al. [65]	2025	Few-shot	Adaptive GNN with semi-supervised multimodal fusion	Image–text feature fusion
Yang et al. [58]	2026	Detection	YOLOv8s-BISW with dedicated stainless-steel-pipe dataset	2490-image, single-class dataset; no independent test set
Xie et al. [60]	2026	Det. + Seg.	ODSL-YOLO: joint detection and segmentation of pipe welds	Task-specific to pressure-pipeline welds

**Table 3:** Representative MFL-based defect detection and quantification works (2018–2026).

Study	Year	Focus	Key Technique/Contribution	Critical Remarks
Ege and Coramik [72]	2018	System	Complete MFL measurement system for pipeline inspection	System and probe design
Peng et al. [12]	2020	Signal analysis	MFL data analysis for pipeline corrosion assessment	Handcrafted features; limited cross-pipeline generalization
Ding et al. [73]	2022	System	MFL system for quantitative identification of broken wires in bridge cables	Task-specific to bridge cables
Long et al. [74]	2022	Probe	Ultra-high-definition MFL topology for crack quantification	Validated on high-aspect-ratio cracks only
Liu et al. [82]	2023	Deep learning	Cascade attention network under complex magnetic backgrounds	Evaluated on in-house inspection data
Liu et al. [81]	2025	Deep learning	Lightweight online weld defect detection for MFL systems	Detection evaluated; sizing outside the reported task
Shen et al. [97]	2024	Quantification	CNN inverse model for corrosion size and location	FE-simulated signals; field evaluation not reported
Xiong et al. [85]	2024	Quantification	Physics-informed neural network for defect size estimation	Synthetic dipole-model data
Park et al. [89]	2025	Deep learning	Semi-supervised learning for MFL-based surface inspection	Gains depend on unlabeled-data quality
Yang et al. [90]	2026	Data augmentation	Diffusion-model generation of synthetic MFL signals	Synthetic-to-real gap not fully characterized
Chen et al. [80]	2026	Detection + sizing	Cascaded YOLOv11 and physics-informed network on MFL data	End-to-end error under field variability not reported
Li et al. [101]	2026	Quantification	Joint quantification integrating magnetic-flux and image data	Image-channel provenance unclear in the accessible record
Wang et al. [87]	2026	Quantification	Physics-guided evolutionary deep learning for defect inversion	Specialized stress–hydrogen coupling setting
Qu et al. [102]	2026	Benchmark	PipeMFL-240K: large-scale public MFL dataset and benchmark	Damage categories remain far below structural components

**Table 4:** Datasets anchoring quantitative comparison in vision-based and MFL-based steel-surface defect detection.

Dataset	Year	Modality	Scale	Classes	Availability
NEU-DET [52,54]	2013	Vision, grayscale 200 × 200	1800 images	6 classes, bounding boxes	Public (Northeastern University)
GC10-DET [44,54]	2020	Vision, grayscale 2048 × 1000	2294 images in the original release; 3570 in the version used by [54] (the difference is not explained in either source)	10 classes, bounding boxes	Public (GitHub)
Severstal [65]	2019	Vision, 1600 × 256	18,074 images (12,568 annotated train/5506 competition test)	4 defect types, boxes/masks	Public (Kaggle)
PipeMFL-240K [102]	2026	MFL images	240,320 main images (144,192/48,064/48,064; approx. 6:2:2 split *within* each pipeline) + 9000-image zero-shot set (unseen pipeline); 200,020 objects total incl. zero-shot portion	4 damage + 8 component classes	Public
GMAW weld set [53]	2023	Vision	7580 images	8 weld-defect classes	Not public
Weld X-ray set [57]	2023	X-ray	3153 images	Weld defects	Not public
STF [58]	2026	Vision, grayscale 1922 × 2592	2490 images (1992 train/498 val)	1 class (crack), bounding boxes	On request; no independent test set (stated by the authors)

**Table 5:** Comparison of fusion strategies for image–MFL inspection, including serial task division as observed in practice. The last column classifies the evidence pairing of the cited instances.

Fusion Level	Definition	Strengths	Limitations	Representative Works	Evidence Pairing
Data-level	Registered raw images and MFL samples fused before modeling	Maximal information retention; end-to-end learning	Highly sensitive to registration error; requires spatially registered, state-consistent measurements	[15,40]	Adjacent: multispectral
Feature-level	Modality-specific embeddings merged by concatenation, gating, or attention	Balances flexibility and performance; compatible with cross-attention	Requires paired samples; fusion weights hard to interpret	[105,106,107]	Adjacent: spectral–visual/multichannel
Decision-level	Independent per-modality decisions combined by rules or evidence theory	Accommodates asynchronous inputs and sensor dropout with explicit missing-modality handling; modular	Low-level cross-modal correlations lost	[14,23,108]	Adjacent: ECT–IRT–MFL/IRT–vision
Serial task division	Modalities handle different sub-tasks in sequence (e.g., vision for recognition, MFL for sizing)	Requires only component/defect association; each stage uses the modality best suited to it	No shared representation; errors propagate downstream	[109]	Camera–MFL: serial workflow [109]

**Table 6:** Proposed fusion-quality evaluation framework for dual-modality inspection, with the measurement status found in the reviewed corpus. Brier scores combine calibration and discrimination. Application-specific acceptance thresholds require validation. “Not reported” means that no study within the search scope reports the quantity; it does not denote missing information.

Metric	What to Report	Why It Matters	Status in Corpus
Registration tolerance	Residual pixel-to-MFL misalignment (mm) vs. fusion gain	Determines whether field registration suffices	Not reported
Gain over best single modality	ΔmAP/accuracy vs. strongest single-modality model on identical samples and splits	Tests added value against a strong single-modality reference	Measured for image–text [65], spectral–visual [105], ECT/thermography/MFL [14]; camera–MFL: not reported
Recall at fixed false-alarm rate	Detection recall at an operationally fixed FP rate	Relates detection to an operational false-alarm budget	Related quantities only [53,65]; fixed-operating-point comparison not reported
Sizing error vs. reference	Depth/width MAE or bias against UT or excavation references	Links detection to integrity grading	Measured for MFL-only [74] and combined diagnostics [100]; camera–MFL: not reported
Missing-modality degradation	Performance with each modality dropped or corrupted	Asynchronous field acquisition loses modalities intermittently	Method precedents [38]; camera–MFL: not reported
Uncertainty calibration	Calibration error and abstention behavior under distribution shift	Grading decisions need trustworthy confidence	MOI–IRT: Brier 0.0271/0.0529 at full/20% training data [108] (publisher record); camera–MFL: not established

**Table 7:** Fusion-related quantitative evidence, grouped by comparison design. Values and source locations are as reported in the original studies; no row pairs an optical camera with MFL. Row (iv) records a fusion failure. ^*a*^ The 5.37-percentage-point few-shot estimate of [65] is excluded: the comparator row is labeled “multi-modal fusion” in the original table, and the four-class dataset versus 5-way task definition does not support attribution to the text modality.

Study	Evidence Type	Modalities	Task	Reported Comparison (Source)	Attribution Limits
Kong et al. [65]	(i) Same-study controlled comparison	Image + text	Few-shot strip-steel classification	93.00% → 96.24% accuracy; +4.00 pp prec.; +3.40 pp rec.; +3.55 pp F1 (their Table 5)	Same splits reported; few-shot estimate excluded ^*a*^
Zhang et al. [105]	(i) Same-study controlled comparison	Spectral + visual	Laser-welding defect classification	Spectral-only vs. visual-only vs. fused classifiers, with backbone ablations	Backbone ablations included
Heideklang and Shokouhi [14]	(i) Same-study controlled comparison	ECT + thermography + MFL	Groove detection on a curved steel bearing shell (15 EDM grooves, 11–354 μm)	Fused decision outperforms single-sensor detection for most grooves; best rules: geometric/harmonic mean, product (their Figure 10, AUC-PR-0.5 metric)	Laboratory EDM grooves; intentionally degraded MFL channel; AUC-PR-0.5 evaluation
Kong et al. [65]	(ii) System-level before/after	Image + text	90-day line deployment	False alarms 2.9% → 0.7%; edge-crack recall 0.78 → 0.83	Baseline CNN vs. complete system; added-text effect not isolated
Tang et al. [16]	(iii) Capability demonstration	MFL + EMAT	Surface + internal defect detection	0.5 mm surface cracks and defects at 16.7% wall depth detected simultaneously	Matched single-modality comparison: not reported
Heideklang and Shokouhi [14]	(iv) Fusion-failure observation	ECT + thermography + MFL	Same specimen as row above	Strict agreement rules (product, geometric/harmonic mean, minimum) misclassify grooves nr. 8–9 as false alarms under inter-sensor conflict (their Section 3.4.1)	Observed rejection of true single-sensor indications

**Table 8:** Reported detection and quantification performance of representative works. Values are as reported by the original papers; mAP@0.5 and mAP@[.5:.95] follow the COCO convention.

Study	Modality	Dataset (Size)	Metric	Reported Value	Note
Guo et al. [52]	Vision	NEU-DET (1800)	mAP@[.5:.95]	75.2% @ 30.6 FPS	+7.0 pp over YOLOv5 baseline (their Table 2); ablation endpoint 75.7% @ 29.1 FPS, +7.5 pp (their Table 3)
Pan et al. [57]	Vision (X-ray)	Self-built weld (3153)	mAP@0.5	92.6% @ 98 FPS	Recall prioritized; deployed on a pipe line
Wang et al. [53]	Vision	Self-built GMAW weld (7580, 8 classes)	mAP@0.5	95.3%	Miss rate 21.47% → 1.68%; params −30.1%
Lu et al. [44]	Vision	NEU-DET/GC10-DET	mAP@0.5	82.3%/72.0%	Lightweight industrial design
Wu et al. [54]	Vision	NEU-DET/GC10-DET	mAP@0.5; mAP@[.5:.95]	83.14%/67.27%; 47.37%/34.09%	Transformer; strict-IoU score much lower
Yang et al. [58]	Vision	Self-built stainless tube (STF, 2490)	mAP@0.5	97.9% @ 3.7 ms/img	+3.3 pp over YOLOv8s; single class; no independent test set
Xiong et al. [85]	MFL sizing	Synthetic dipole-model data	Relative accuracy	Improved vs. NN/SVM	Physics-informed; fewer physical violations
Long et al. [74]	MFL sizing	FE simulation + physical experiments	Sizing error	Depth ≤ 0.7 mm; width ≤ 0.1 mm	Crack aspect ratio > 4; MFSI spatial-integration method
Qu et al. [102]	MFL imaging	PipeMFL-240K (240,320 main imgs + 9000 zero-shot; 200,020 objects incl. zero-shot)	mAP@0.5	0.498 (best: YOLOv5-l, YOLOv8-x-world-v2)	Damage classes AP50 < 0.3 vs. best component classes (valves, sleeves) > 0.8

**Table 9:** Candidate acquisition configurations for curved steel components, with applicability conditions and evidence limits. “Serial” denotes sequential task division with component/defect association; “registered fusion” denotes joint modeling of spatially aligned pairs. Evidence levels: [direct] = measured in the reviewed corpus; [adjacent] = measured for neighboring modality pairings or tasks; [hypothesis] = unvalidated design reasoning.

Inspection Condition	Candidate Configuration	Applicability Conditions and Evidence Limits
Manufacturing-stage strip/plate; controlled illumination; surface-only defects (cracks, scratches, pits); detection or localization output	Vision alone	Public benchmarks reach 82–83% mAP@0.5 [direct for detection accuracy on curated data [44,54]]. Vision-only inspection may be appropriate when optical access and the required detection and localization performance have been demonstrated for the intended defects; verify the outputs required by the inspection task, since benchmark mAP alone does not establish size-specific detection probability, operating-point recall, or depth-sizing performance [hypothesis for sufficiency]
In-service pipeline; coated or contaminated surface; metal loss and near-surface anomalies; component mapping along the line	MFL alone (ILI tooling)	Vision is blind under coatings; MFL is the established in-service modality [direct [9,102]], with quantification as the open bottleneck [74,85]
Curved steel surfaces (tanks, gates, hulls) with rust or occlusion, where both surface morphology and near-surface sizing matter	Serial task division: vision for recognition/triage, MFL for confirmation and sizing	A serial camera–MFL workflow is reported in [109] [direct]. Asynchronous acquisition still requires component/defect association; restricting MFL to vision-positive regions leaves vision-missed defects uncovered [hypothesis for general sufficiency]
Safety-critical grading of compound defects, where a quantified reliability gain over the best single modality must be demonstrated	Registered fusion, only if paired acquisition and a registration error budget are in place	Adjacent registration evidence [14] [adjacent]; camera–MFL reliability gain not shown in the reviewed corpus (Table 7) [hypothesis]
Field acquisition by a climbing robot plus a separate MFL device at different times	Separate registered passes; fusion level chosen after verifying spatial alignment and measurement-state consistency	Velocity and lift-off variability affect the MFL response and localization; separate passes reduce mounting constraints but require spatial registration and state-consistent measurements [adjacent [17,119]; hypothesis for the configuration choice]
Conditions where one modality is intermittently unavailable (illumination failure, skipped MFL pass)	Fusion with graceful degradation, or single-modality fallback	Test sensor removal, corruption, and conflicting indications; specify single-modality fallback and conflict-handling rules (Table 6) [adjacent [14]; hypothesis]

## Data Availability

No new experimental data were created in this review. The per-work extraction table of the literature corpus is provided as Table S1 in the Supplementary Materials. Figure 1, Figure 2, Figure 3, Figure 4, Figure 5 and Figure S2 were drawn by the authors with Python 3.9.6 (Matplotlib 3.9.4) scripts from Table S1 and the values reported in the cited studies; these scripts are provided as Code S1 in the Supplementary Materials. The photographs in Figure S1 were taken by the authors of their own test platform. No generative AI tool was used to create or edit any figure.

## Supplementary Materials

The following supporting information can be downloaded at: https://www.mdpi.com/article/10.3390/s26216850/s1, Supplementary Note S1: full database-specific search strings, the 16 Google Scholar queries, the October 2026 query extension, search dates, deduplication method, and stage-by-stage screening counts; Table S1: per-work extraction table for all 126 reviewed works: document type, object material, sensing modalities, core/adjacent classification, manufacturing vs. in-service stage, curved geometry, whether fusion is actually performed, whether paired acquisition is used, and whether a quantitative comparison is reported; Figure S1: photographs of the authors’ planned acquisition testbed; Figure S2: distribution of publication venues and journal quartiles of the reviewed corpus; Figure S3: screenshots of the Web of Science and IEEE Xplore result pages with search strings and record counts; Code S1: Python scripts used to draw Figure 1, Figure 2, Figure 3, Figure 4, Figure 5 and Figure S2.

## Abbreviations

The following abbreviations are used in this manuscript:

MFL	Magnetic flux leakage
NDT/NDE	Nondestructive testing/evaluation
ECT	Eddy current testing
EMAT	Electromagnetic acoustic transducer
UT	Ultrasonic testing
CNN	Convolutional neural network
LSTM	Long short-term memory
YOLO	You Only Look Once
ROI	Region of interest
PINN	Physics-informed neural network
JCR	Journal Citation Reports
