# Supplementary material for: Toward Image–Magnetic Flux Leakage Dual-Modality Inspection of Steel Pipes and Curved Steel Surfaces: Evidence, Fusion Strategies, and Robotic Acquisition
Source: Sensors (Basel). 2026 Oct 29;26(21):6850. doi: 10.3390/s26216850 (PMC13766398; doi:10.3390/s26216850)
Supplement: Supplementary file 1 [file sensors-26-06850-s001.zip › Figure_S3_Search_Screenshots.pdf]

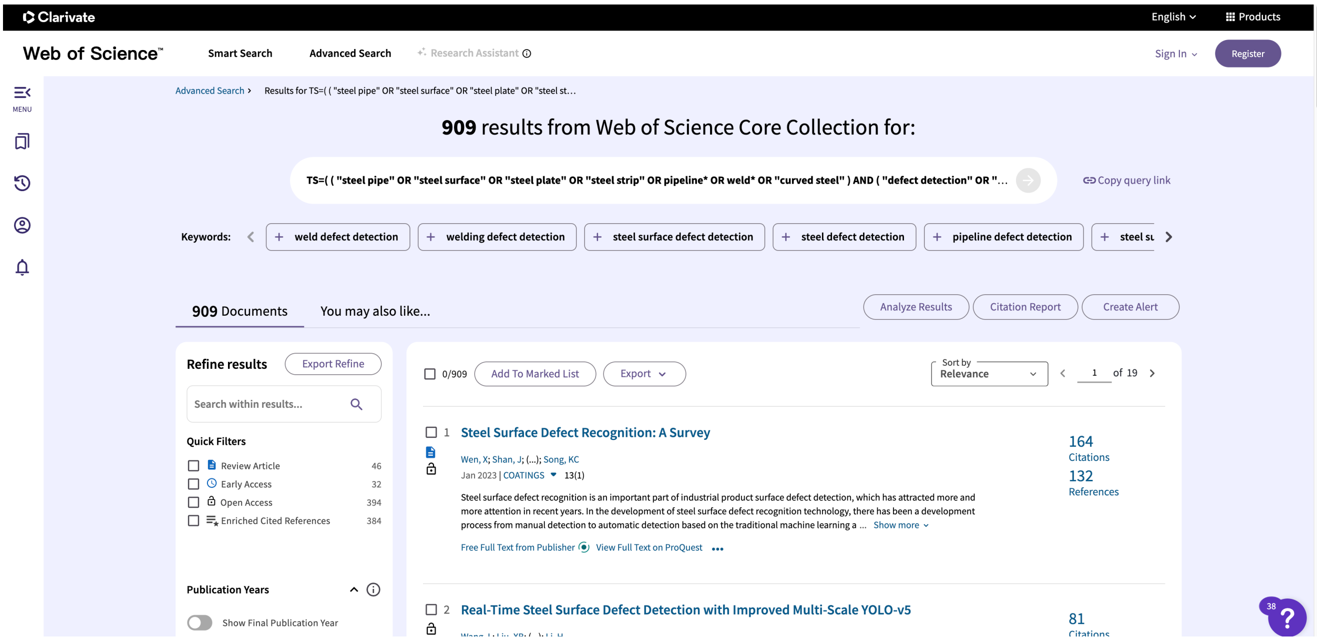

(a)

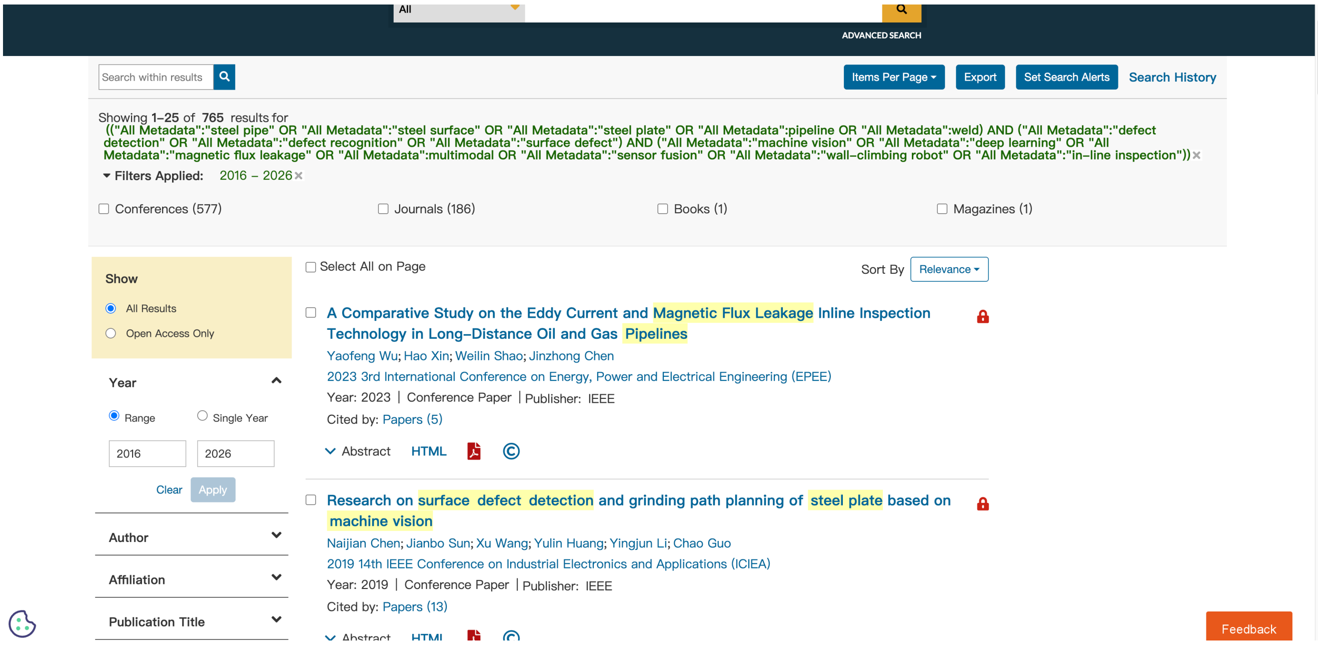

(b)

Figure S3. Result pages of the primary database searches executed on 7 October 2026. (**a**) Web of Science Core Collection (SCIE), topic search: 909 records. (**b**) IEEE Xplore, Command Search on All Metadata with the 2016–2026 year filter: 765 records. The complete search strings are given in Supplementary Note S1, Section 2.
