# Supplementary material for: Toward Image–Magnetic Flux Leakage Dual-Modality Inspection of Steel Pipes and Curved Steel Surfaces: Evidence, Fusion Strategies, and Robotic Acquisition
Source: Sensors (Basel). 2026 Oct 29;26(21):6850. doi: 10.3390/s26216850 (PMC13766398; doi:10.3390/s26216850)
Supplement: Supplementary file 1 [file sensors-26-06850-s001.zip › Supplementary_Note_S1.pdf]

Supplementary Note S1: Search Protocol and Screening Log

Manuscript: sensors-4624955. Toward Image–Magnetic Flux Leakage Dual-Modality Inspection of Steel Pipes and Curved Steel Surfaces: Evidence, Fusion Strategies, and Robotic Acquisition

This note documents the full search protocol behind Figure 3 (PRISMA-style flow) of the revised manuscript.

1. Search block logic (shared across databases)

- **Object block O:** "steel pipe" / "steel surface" / "steel plate" / "steel strip" / pipeline* / weld* / "curved steel"
- **Defect block D:** "defect detection" / "defect recognition" / "flaw detection" / "surface defect*"
- **Technique block T:** "machine vision" / "deep learning" / "magnetic flux leakage" / "MFL" / multimodal* / "sensor fusion" / "data fusion" / "wall-climbing robot" / "climbing robot" / "in-line inspection"
- **Time window:** January 2016 – October 2026. **Language:** English (Chinese-language literature was covered through the four Chinese Google Scholar queries).

2. Database-specific search strings and settings

Primary databases: Web of Science Core Collection (SCIE) and IEEE Xplore. Supplementary source: Google Scholar.

Web of Science Core Collection, SCIE (executed 7 October 2026; 909 records):

```
TS=( ( "steel pipe" OR "steel surface" OR "steel plate" OR "steel strip" OR pipeline* OR weld* OR "curved
steel" ) AND ( "defect detection" OR "defect recognition" OR "flaw detection" OR "surface defect*" ) AND (
"machine vision" OR "deep learning" OR "magnetic flux leakage" OR "MFL" OR multimodal* OR "sensor fusion"
OR "data fusion" OR "wall-climbing robot" OR "climbing robot" OR "in-line inspection" ) ) AND PY=(2016-
2026) AND LA=(English)
```

Export: Full Record including abstracts.

IEEE Xplore (executed 7 October 2026; 765 records; Command Search, All Metadata):

```
("All Metadata":"steel pipe" OR "All Metadata":"steel surface" OR "All Metadata":"steel plate" OR "All
Metadata":pipeline OR "All Metadata":weld) AND ("All Metadata":"defect detection" OR "All
Metadata":"defect recognition" OR "All Metadata":"surface defect") AND ("All Metadata":"machine vision" OR
"All Metadata":"deep learning" OR "All Metadata":"magnetic flux leakage" OR "All Metadata":multimodal OR
"All Metadata":"sensor fusion" OR "All Metadata":"wall-climbing robot" OR "All Metadata":"in-line
inspection")
```

Year filter 2016–2026 applied in the interface. Export: citations with abstracts.

Screenshots of the Web of Science and IEEE Xplore result pages, showing the search strings and record counts, are provided as Figure S3.

Google Scholar: supplementary source (executed 28 September 2026). Google Scholar does not support long nested Boolean strings, so 16 short keyword queries were issued (12 English, interface language en; 4 Chinese, interface language zh-CN; each Chinese query is given below with its English translation), each restricted to publication years 2016–2026. For every query the 50 top-ranked records by Google Scholar relevance were exported (16 × 50 = 800 records). Google Scholar displays only an approximate hit count per query; these counts were not recorded. The 800 records are therefore a relevance-ranked sample, not the full retrieval of each query, and the reproducible core of the search is the Web of Science and IEEE Xplore component. Raw exports (one CSV per query, titles, authors, venues, years) are archived by the authors.

#	Query ID	Query string	Language	Records
1	en_visual_1	steel surface defect detection deep learning	English	50
2	en_visual_2	steel pipe surface defect detection YOLO	English	50

#	Query ID	Query string	Language	Records
3	en_visual_3	transformer surface defect detection industrial	English	50
4	en_visual_4	few-shot surface defect detection deep learning	English	50
5	en_mfl_1	magnetic flux leakage testing pipeline defect	English	50
6	en_mfl_2	magnetic flux leakage signal deep learning	English	50
7	en_mfl_3	eddy current testing defect deep learning	English	50
8	en_fusion_1	multimodal fusion nondestructive testing defect	English	50
9	en_fusion_2	multimodal image signal fusion deep learning inspection	English	50
10	en_fusion_3	visual electromagnetic sensor fusion defect detection	English	50
11	en_robot_1	wall climbing robot inspection steel	English	50
12	en_robot_2	magnetic climbing robot weld defect inspection	English	50
13	zh_visual_1	Chinese query 钢管表面缺陷检测 深度学习 (English translation: steel pipe surface defect detection, deep learning)	Chinese	50
14	zh_mfl_1	Chinese query 漏磁检测 缺陷识别 深度学习 (English translation: magnetic flux leakage testing, defect recognition, deep learning)	Chinese	50
15	zh_fusion_1	Chinese query 多模态融合 缺陷检测 无损检测 (English translation: multimodal fusion, defect detection, nondestructive testing)	Chinese	50
16	zh_robot_1	Chinese query 爬壁机器人 无损检测 焊缝 (English translation: wall-climbing robot, nondestructive testing, weld)	Chinese	50
		**Total**		**800**

Scopus. Not searched, because institutional access was unavailable.

Targeted query extension (executed 12 October 2026). The initial strings above privilege "defect detection/recognition" phrasing in the defect block. An extended WoS SCIE topic query added "inspection" and "sizing" to the defect block (its technique block is shorter than that of the initial query), and the title-level exclusions of the initial searches were re-checked for review-type records. The record count of the extension query was not recorded; only the surveys added to the corpus are reported.

```
TS=( ( "steel pipe" OR "steel surface" OR "steel plate" OR "steel strip" OR pipeline* OR weld* OR "curved steel" ) AND ( "defect detection" OR "defect recognition" OR "surface defect*" OR "inspection" OR "sizing" ) AND ( "magnetic flux leakage" OR "MFL" OR "machine vision" OR multimodal* OR "sensor fusion" OR "wall-climbing robot" OR "in-line inspection" ) ) AND PY=(2016-2026) AND LA=(English)
```

Four surveys were added. One was identified by the extension query: Qiu et al. 2026 (Sens. Actuators A Phys. 398, 117367), whose title contains "MFL inspection" and therefore matched no initial defect-block term. Three were recovered by the re-check of title-level exclusions: Ma et al. 2025 (Sensors 25, 4873), Xu et al. 2026 (J. Pipeline Sci. Eng. 6, 100356), and Khalid et al. 2026 (Adv. Eng. Inform. 76, 104978); these were present in the 7 October export but had been excluded at title level because their titles contain no "defect" term. All four are included in the corpus and in Table 1 of the manuscript.

3. Merging, deduplication, and screening counts

Stage	Count	Method
Records identified	2474	909 (WoS) + 765 (IEEE Xplore) + 800 (Google Scholar, 16 queries × top 50)
Duplicates removed	308	DOI primary key + normalized-title matching
Unique records screened (title/abstract)	2166	Inclusion: steel/ferromagnetic object AND defect detection/inspection AND vision/electromagnetic/fusion/robotic method, 2016+
Excluded at title/abstract	1968	Off-topic after keyword relevance screening
Full-text assessed	198	Full-text eligibility check
Excluded at full text	76	72 off-scope relative to the inclusion criteria or lacking a described method and evaluation (e.g., abstract-only records, product notes; the experiment-reporting requirement applied to records claiming original research; reviews/surveys and the adjacent-domain works of Section 4 were retained via a separate background and positioning channel); 4 incomplete bibliographic records
Included (main searches)	122	35 vision, 41 MFL/electromagnetic, 30 fusion, 16 robotic platforms
Added on 12 October 2026	+4	Extension query 1 (Qiu 2026); re-check of title-level exclusions 3 (Ma 2025, Khalid 2026, Xu 2026). Surveys, by theme: MFL/electromagnetic +2 (Qiu 2026; Ma 2025); fusion +1 (Khalid 2026); robotic platforms +1 (Xu 2026); vision +0
**Final corpus**	**126**	35 vision, 43 MFL/electromagnetic, 31 fusion, 17 robotic platforms; 104 research articles + 22 reviews/surveys; 95 English + 31 Chinese

Theme totals reconcile as 35 + 0 = 35 (vision), 41 + 2 = 43 (MFL/electromagnetic), 30 + 1 = 31 (fusion), and 16 + 1 = 17 (robotic platforms); theme assignments follow the Theme column of Table S1.

Screening was performed by the first author; uncertain records were discussed with the corresponding author. The per-work extraction table is provided as Table S1, which includes a document-type column (research article vs. review/survey) and a Summary sheet with the object-by-modality counts.

4. Scope bookkeeping

Of the 126 included works, **111** address steel or other ferromagnetic components directly and form the **core corpus**; **15** serve as **adjacent-domain evidence** in two categories:

- **Adjacent-A (9 works):** the inspection object is entirely non-ferromagnetic: food and plant monitoring (2), tire and building inspection (2), composite and friction-stir-weld testing (3), and concrete-structure robots with visual or hammering inspection (2). These works contribute transferable fusion, registration, or robotic-carrier methods but no ferromagnetic inspection evidence.
- **Adjacent-B (6 works):** cross-domain methodological precedents: cross-material fatigue prediction, general incomplete-modality learning, multimodal ultrasonic sensor fusion, laser-thermography fusion, general multimodal perception, and broad NDT image fusion. These works supply method templates (e.g., missing-modality training, fusion architectures) rather than domain evidence.

Both categories are cited only where they offer transferable fusion, registration, or missing-modality methods and are flagged as adjacent-domain evidence at the point of citation. Table S1 marks each work as "Core", "Adjacent-A", or "Adjacent-B" together with object material, sensing modalities, stage, geometry, and evidence-type fields; cells that cannot be determined reliably from the record are marked "unclear". A separate "Pairing evidence" column distinguishes direct pairing evidence (the

serial camera–MFL workflow of Gao et al.), the unresolved candidate (Li et al., image channel unconfirmed), adjacent non-camera–MFL pairings, and non-fusion works (n/a), so that corpus-scope labels (core/adjacent) are not conflated with fusion-pairing labels (direct/adjacent).

5. Quartile assignment rule (Figure S2)

Quartile labels in Figure S2 follow the Journal Citation Reports release current at the time of collection (JCR 2025, released June 2026), using the journal's quartile in the category most relevant to this review's topic (typically "Instruments & Instrumentation" or "Engineering, Multidisciplinary"); for journals listed in multiple categories, the best quartile is reported, and the rule is applied uniformly to all 95 English-language works. Quartiles describe where the literature is published; they are not used as evidence of methodological quality or field maturity, and Figure S2 is a description of this collected corpus, not a field-wide growth or quality estimate. Chinese-language journals, conference papers, preprints, journals without a Journal Impact Factor, and one journal removed from SCIE are grouped under "Q4 / conf. / other".

6. Pairing-evidence counts (Section 5.6)

Within the fusion theme of the corpus (31 works, including one pipeline-damage survey admitted as background): 1 implements a serial optical–MFL workflow (Gao et al.); 1 is a candidate whose image channel cannot be confirmed as independent optical acquisition (Li et al., IEEJ TEE 2026); the remainder involve adjacent modality pairings (MOI–IRT; ECT/thermography/MFL; magneto-optical imaging; MFL–EMAT; spectral–visual; image–text) or cross-domain methodological precedents. Registered optical–MFL fusion quantified against matched single-modality baselines: 0 within the search scope.

7. Figure S1: Planned acquisition testbed

Figure S1 shows the authors' planned testbed for asynchronous dual-modality acquisition: (a) close-up of the magnetic-adhesion wall-climbing robot with its camera, illumination, and magnetic wheels; (b) the robot operating on a vertical wall with illumination on; (c) local view of the specimen showing artificial scratches and a circumferential weld with the robot in place; (d) overall view of the annular steel plate specimen with distributed prefabricated defects. Paired MFL measurements are not yet available; the specimen's controlled defects and known geometry are intended for measuring the registration error budget discussed in Section 6.3 of the manuscript. Photographs taken by the authors.
