# Supplementary material for: Toward Image–Magnetic Flux Leakage Dual-Modality Inspection of Steel Pipes and Curved Steel Surfaces: Evidence, Fusion Strategies, and Robotic Acquisition
Source: Sensors (Basel). 2026 Oct 29;26(21):6850. doi: 10.3390/s26216850 (PMC13766398; doi:10.3390/s26216850)
Supplement: Supplementary file 1 [file sensors-26-06850-s001.zip › Figure_S1_Planned_Testbed.pdf]

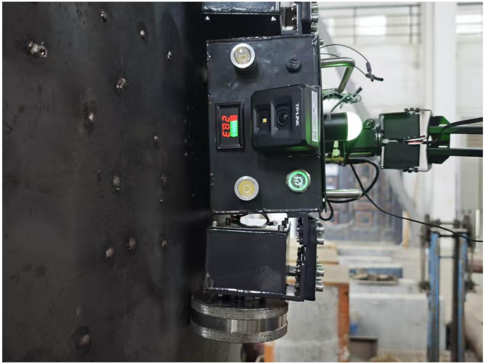

(a)

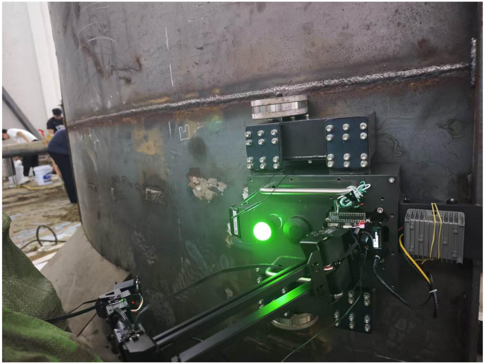

(b)

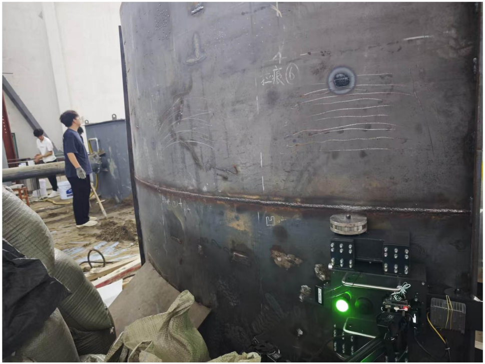

(c)

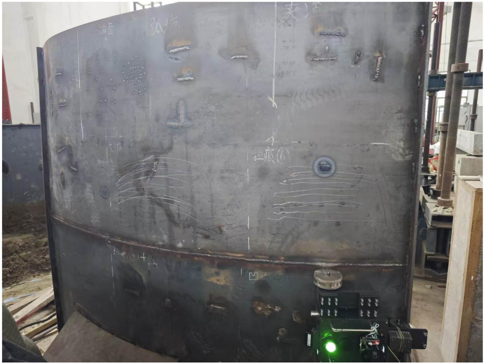

(d)
